# 20 years translation of ultrasonic elastography: technology innovation and clinical applications

**DOI:** 10.1186/1479-5876-10-S2-A30

**Published:** 2012-10-17

**Authors:** Hairong Zheng, Congzhi Wang, Yang Xiao, Yang Shen

**Affiliations:** 1 Paul C. Lauterbur Research Center for Biomedical Imaging, Shenzhen Institutes of Advanced Technology, Chinese Academy of Sciences, China

## 

Stiffness change of tissue is often seen with the progression of pathology. In the past two decades, ultrasonic elastography has emerged as a powerful complementary technique to B-mode ultrasonic imaging, in which strain of target tissues can be imaged and their stiffness can be assessed. The physical principle of elastography is, when deformation is generated in the tissue by an external or internal mechanical stimulus, coherent acoustic echoes can be tracked by a pulse-echo system, then induced strain inside the tissue can be estimated and the relative mapping of strain can be imaged. In addition, the viscoelastic properties of the tissue can be assessed if the mechanical wave velocity propagating in the tissue was measured. Now this technique has been developed to be more flexible and accurate. The qualitative results presented by quasi-static elastogram have changed to numerical quantification of viscoelastic modulus achieved by other innovative methods, such as sonoelastography, transient elastography, acoustic radiation force impulse imaging (ARFI), shear-wave dispersion ultrasound vibrometry (SDUV) and supersonic shear imaging (SSI). Aiming for different tissue or organs, these methods can achieve viscoelasticity assessments on various target objects with sizes from tens of millimeters to several microns. Short or trains of mechanical or acoustic radiation force impacts have been used to generate displacements with different patterns. The induced deformation in the tissue has also been tracked by different algorithms based on both cross-correlation and texture matching approaches. Some of these techniques have been studied by our group, and the combination of ultrasonic elastography and particle imaging velocimetry (PIV) using microbubble contrast agents has been also explored. Ultrasonic elastography has been verified on many normal/abnormal tissues and now widely used in clinical practice, such as on breast disease (fibroadenomas, cysts and cancers), liver fibrosis and cirrhosis, dermatology (melanomas and scars), cardiovascular disease (cardiac muscle disease and arteriosclerosis), musculoskeletal studies, minimally invasive surgery and hyperthermia therapy (temperature monitoring and lesion detection in HIFU) . During these 20 years, ultrasonic elastography has been developed to be a powerful adjunctive technique to traditional medical imaging methods and expected to become an excellent tool for clinical diagnosis and treatment.

